# Embryonic germ cell extracts erase imprinted genes and improve the efficiency of induced pluripotent stem cells

**DOI:** 10.1038/s41598-018-29339-0

**Published:** 2018-07-19

**Authors:** Jing Hu, Qiaoshi Zhao, Yukuan Feng, Na Li, Yanli Gu, Ruizhen Sun, Lian Duan, Yanshuang Wu, Zhiyan Shan, Lei Lei

**Affiliations:** 1 Department of Histology and Embryology, Harbin Medical University, Harbin, 150081 P. R. China; 2 Department of Histology and Embryology, Mudanjiang Medical University, Mudanjiang, 157011 P. R. China; 3 Key Laboratory of Tumor Prevention and Treatment of Heilongjiang Province, Mudanjiang Medical University, Mudanjiang, 157011 P. R. China

## Abstract

Patient-specific induced pluripotent stem cells (iPSCs) have the potential to be useful in the treatment of human diseases. While prior studies have reported multiple methods to generate iPSCs, DNA methylation continues to limit the totipotency and reprogramming efficiency of iPSCs. Here, we first show the competency of embryonic germ cells (EGCs) as a reprogramming catalyst capable of effectively promoting reprogramming induced by four defined factors, including Oct4, Sox2, Klf4 and c-Myc. Combining EGC extracts with these four factors resulted in formation of more embryonic stem cell-like colonies than did factors alone. Notably, expression of imprinted genes was higher with combined induction than with factors alone. Moreover, iPSCs derived from the combined inductors tended to have more global hypomethylation. Our research not only provides evidence that EGC extracts could activate DNA demethylation and reprogram imprinted genes, but also establishes a new way to enhance reprogramming of iPSCs, which remains a critical safety concern for potential use of iPSCs in regenerative medicine.

## Introduction

Induced pluripotent stem cells (iPSCs) represent a monumental scientific breakthrough in stem cell biology and regenerative medicine^1,2^, capable of breaking down various ethical and logistical obstacles associated with human embryonic stem cell (ESC) research^3,4^. iPSCs are generated by inducing the four “Yamanaka” transcription factors Oct4, Sox2, Klf4 and c-Myc (OSKM) into somatic cells^5,6^; and essentially, reprogramming is an epigenetic process for changing the fate of cells^7–9^. It involves a number of different mechanisms to overcome the epigenetic barriers that are imposed during differentiation^10–12^.

DNA methylation is a major handicap to reprogramming, causing both low efficiency of somatic cell reprogramming and instability of resulting pluripotent cells^13,14^. Previous studies have shown that differentiation-induced *de novo* DNA methylation can repress a large set of pluripotency genes including Oct4 and Nanog; whereas, active DNA demethylation is required for reactivation of pluripotency gene^15–17^. Furthermore, treatment of somatic cells with compounds that promote DNA demethylation facilitates the complete conversion of partially reprogrammed cells that would otherwise fail to reprogram into a pluripotent state^11,14^. Collectively, this research indicates that by interfering with repressive mechanisms, i.e. DNA methylation, the efficiency of transcription factor-induced reprogramming can be improved^18,19^.

Notably, DNA demethylation appears to be responsible for an increase in the pluripotency of extract-treated cells^20–22^. Reprogramming using extracts involves reversible permeabilization of somatic cells followed by exposure to extracts. Using this approach, several pluripotent cell types, including ESCs^23–26^ and embryonal carcinoma cells^23–27^, have been shown to elicit changes in the cell fate of somatic cells. Indications of reprogramming in this system include induction markers of pluripotency and downregulation of lamin A. More importantly, OCT4 activation is associated with DNA demethylation in the OCT4 promoter^23^; the NANOG promoter appears to be more readily demethylated, because Nanog overcomes reprogramming barriers and induces pluripotency in minimal conditions^28^. Observed alterations in the expression profiles of reprogrammed cells imply epigenetic modifications on DNA have taken place. Nevertheless, demethylation is incomplete and not all regions examined on OCT4 are equally demethylated^29,30^, in contrast to what is seen in ESCs or carcinoma cells. In the mouse embryos, migrating primordial germ cells (PGCs) reach the gonads at around 10.5 dpc. They undergo an extensive active genome-wide DNA demethylation, including erasure of genomic imprints. This rapid demethylation process is complete by 13.5 dpc^31–33^. Derived from PGCs, embryonic germ cells (EGCs) are pluripotent and harbor an epigenome similar to that of PGCs^34,35^. Studies have shown that EGC–thymocyte hybrids induce pluripotency markers and can differentiate into all three germ layers in chimera, which are characterized by demethylation of several non-imprinted and imprinted genes^36^. Furthermore, EGCs contain a substance with discrete roles in cell-fuse-mediated pluripotent reprogramming and imprint erasure in somatic cells^37,38^.

Genomic imprinting is an epigenetic alteration through which gene expression is regulated in a monoallelic manner. Abnormal expression of imprinted genes disrupts fetal development and is associated with both genetic diseases and malignancies^39,40^. Aberrant expression of imprinted genes has been observed with reprogramming of somatic cells by nuclear transfer^41,42^ or viral-mediated factors^43–45^. The methylation abnormalities in these cells result from the incomplete reprogramming. EGC fusion reportedly resets the epigenetic reprogramming of both imprinted and non-imprinted genes, which supports full reprogramming^36^. Yet, the precise mechanism affecting reprogramming remains unclear.

Based on the studies outlined above, we speculate that EGC extracts could enhance reprogramming by its unique capacity to actively drive the DNA demethylation process; however, the exact degree of reprogramming is unclear. Thus, we examined the reprogramming ability and mechanism of EGC extracts, which may have the potential to provide highly efficient and safe iPSCs.

## Results

### Treatment with EGC extracts enhanced generation of iPSCs

**Figure 1:**
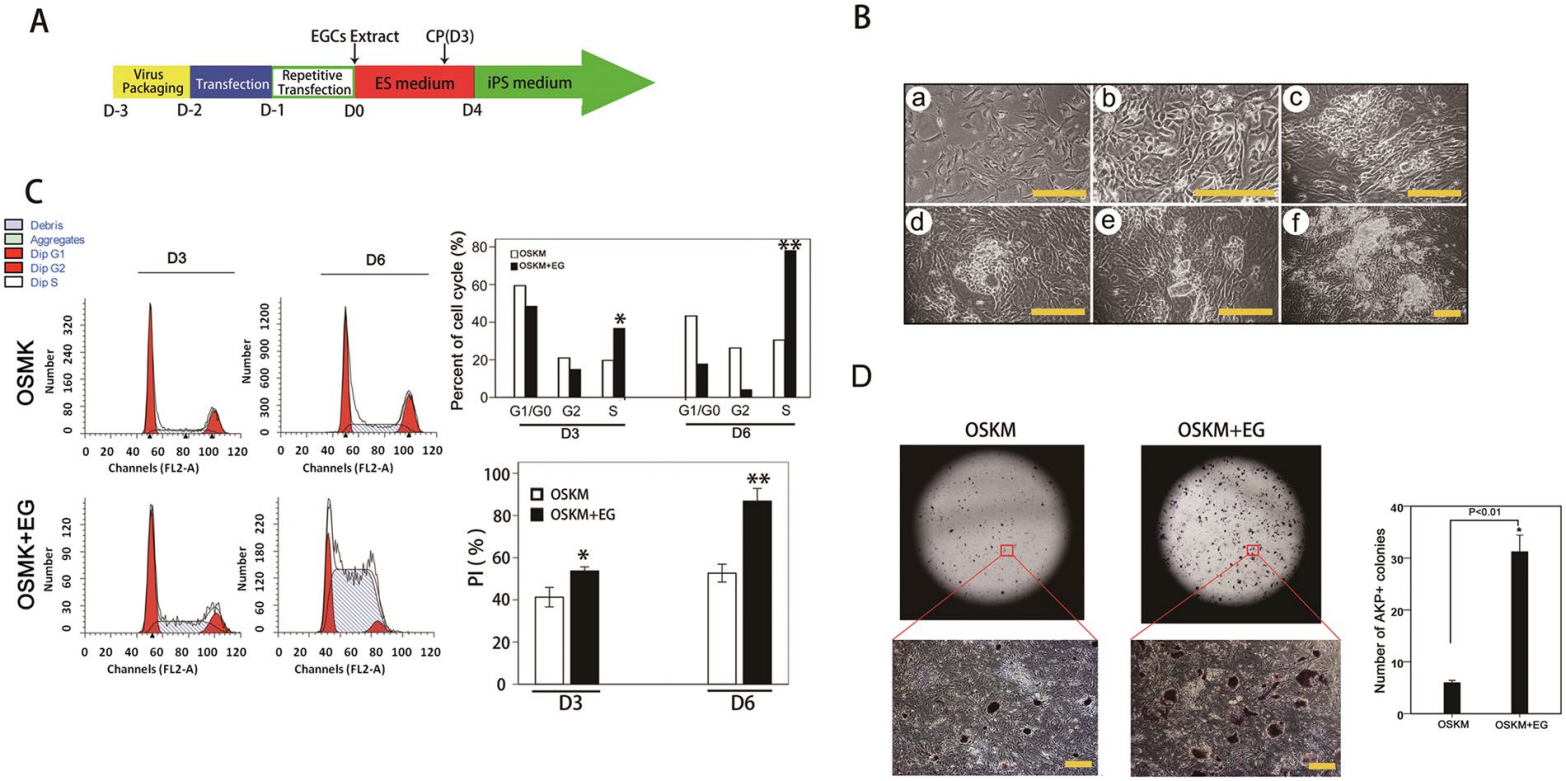
Treatment with embryonic germ cell (EGC) extracts enhances the generation of induced pluripotent stem cells (iPSCs). (**A**) Schematic representation of iPSC generation from mouse embryonic fibroblasts (MEFs) by ectopic expression of Oct4, Sox2, Klf4 and c-Myc (OSKM) followed by exposure to EGC extracts. (**B**) Sequential morphology of iPSC generation with EGC extract treatments (a) of MEFs (b–d) Morphology of epithelium-like cells after infection at day 3 (e) embryonic stem cell-like colonies formed at day 5 (f) and as colonies become bigger at day 7. (**C**) Left: Cell cycle by FACS at day 3 and 6 from four factor-infected MEFs treated with EGC extracts and four factor-infected MEFs. Right: Percentage of cells in each cell cycle phase and PI (cell cycle proliferation index) in OSKM transfected MEFs with or without EGC extracts treatment. Mean values ± standard error of the mean (SEM) of three independent experiments are shown. (**D**) Left: Representative images of alkaline phosphatase-positive (AKP + ) colonies. Cells were fixed at day 8. Right: Number of AKP + colonies. Mean values ± SEM of a representative experiment are shown, n = 3. Scale bar = 100 µm.

To investigate the role of EGC extracts in reprogramming, we first established EGCs from E12.5 embryos such that EGCs closely resembled ESCs with regard to pluripotency and related marker expression (Supporting Information Fig. S1). Next, we examined the effect of EGC extracts on MEFs infected with the four reprogramming factors. As described in Fig. 1A, the induction process involves infection of MEFs with retroviruses encoding the four factors with or without EGC extracts for 1 h and culture in ESC medium thereafter. As shown by FACS (Fig. 1C), percentage of S phase cells (36.7%, 78.11%) in the combined group (EGC extract plus OSKM treatment) was remarkably higher than that in the group without EGC extract treatment (19.68%, 30.48%) on 3 and 6 days after treatment. Proliferation activity [cell cycle proliferation index, PI (%)] of cells in the combined group was higher than that observed in the group without EGC extract treatment. Further, the morphology and growth of cells changed quickly (Fig. 1B). iPSC colonies were observed in the combined group first (at day 5) and the number of colonies increased with time in culture; whereas, iPSC colonies could only be readily identified in the four-factor group at day 7. Moreover, the number of AKP-positive colonies in the combined group was 5–7 times higher than in the four-factor treated group (Fig. 1D). As previously reported, an ESC-like morphology was used to identify putative iPSC colonies and the efficiency was roughly determined by number of AKP-positive colonies; thus, our results show that treatment with EGC extracts enhances four-factor mediated reprogramming.

### iPSCs induced by both EGC extracts and four factors are pluripotent

**Figure 2:**
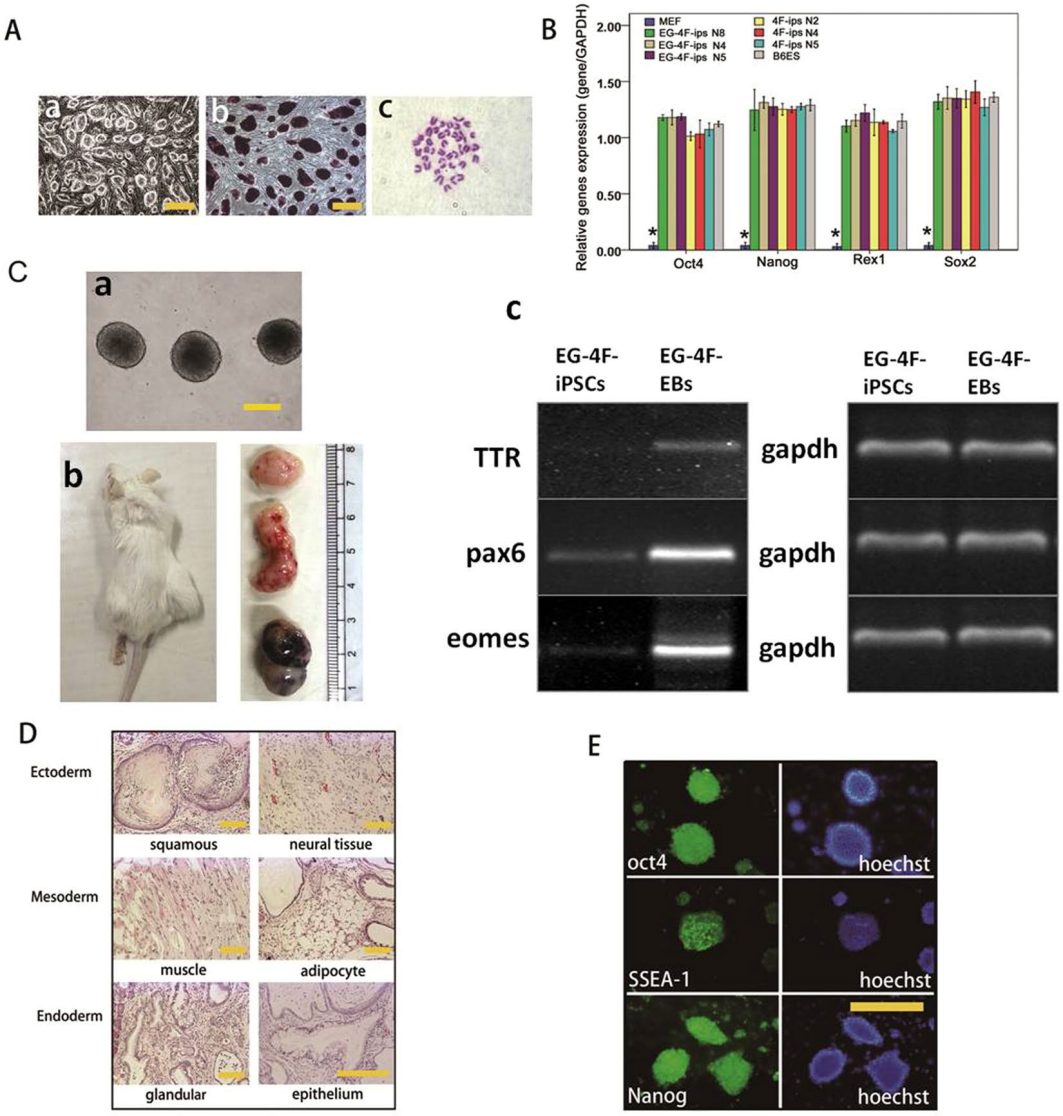
Expression of pluripotent genes, *In vitro* and *in vivo* differentiation and the of iPSCs generated by EGC extract and OSKM combination. (**A**-a) Morphology of EG-4F-iPSCs P15. (**A**-b) Alkaline phosphatase-positive colonies and (**A**-c) normal karyotype (20pairs). (**B**) Quantitative PCR analysis of pluripotent markers Oct4, Nanog, Rex1 and Sox2 in EG-4F-iPSCs. Error bars indicate standard error of the mean (n = 3). Results were normalized to GAPDH expression. *P < 0.01, versus other cells. (**C**-a) Embryoid body formation by suspension culture. (**C**-b) Teratomas. (**C**-c) RT-PCR analysis of germ layer markers. The grouping blots of germ layer markers were cropped from different parts of the same gel. The grouping blots of gapdh were cropped from different parts of the same gel. (**D**) Hematoxylin and eosin staining of teratomas. Scale bar = 50 µm. (**E**) Immunofluorescence staining of pluripotent genes.

iPSCs were generated in both the combined and four-factor groups, named EG-4F-iPSCs and 4F-iPSCs, respectively. EG-4F-iPSCs exhibited strong AKP activity (Fig. 2A-b) and expressed pluripotency markers, such as Oct4 and SSEA1 (Fig. 2E), in immunofluorescence analysis. Karyotype analysis exhibited normal 40 XX chromosomes (Fig. 2A-c). Real-time PCR analysis indicated that expression levels of pluripotency marker genes in EG-4F-iPSCs, including Oct4, Nanog, Sox2 and Rex1, were similar to those of 4F-iPSCs and markedly elevated compared with levels in MEFs (Fig. 2B).

We also examined the differentiation capacity of EG-4F-iPSCs *in vitro* and *in vivo* (Fig. 2C,D). Similar to 4F-iPSCs, EG-4F-iPSCs form EBs in suspension culture and EBs expressed the three germ layer markers, Pax6 (ectoderm), eomes (mesoderm), and TTR (endoderm), as seen by RT-PCR (Figs 2C and S3). Further, EG-4F-iPSCs injected into nude mice formed teratomas after 6 weeks. Histological examination of resulting teratomas revealed tissues originating from all three embryonic germ layers. Taken together, these results indicate there is no significant difference in self-renewal or differentiation capacity between EG-4F-iPSCs and 4F-iPSCs.

### EGC extracts promoted DNA demethylation in OSKM treated cells

It has been suggested that epigenetic reprogramming might be necessary for the embryonic genome to return to a pluripotent state. As such, we were interested in comparing global methylation patterns between EG-4F-iPSCs and 4F-iPSCs. Global MeDIP analysis allowed for identification of the baseline between methylated and unmethylated sequences.

**Figure 3:**
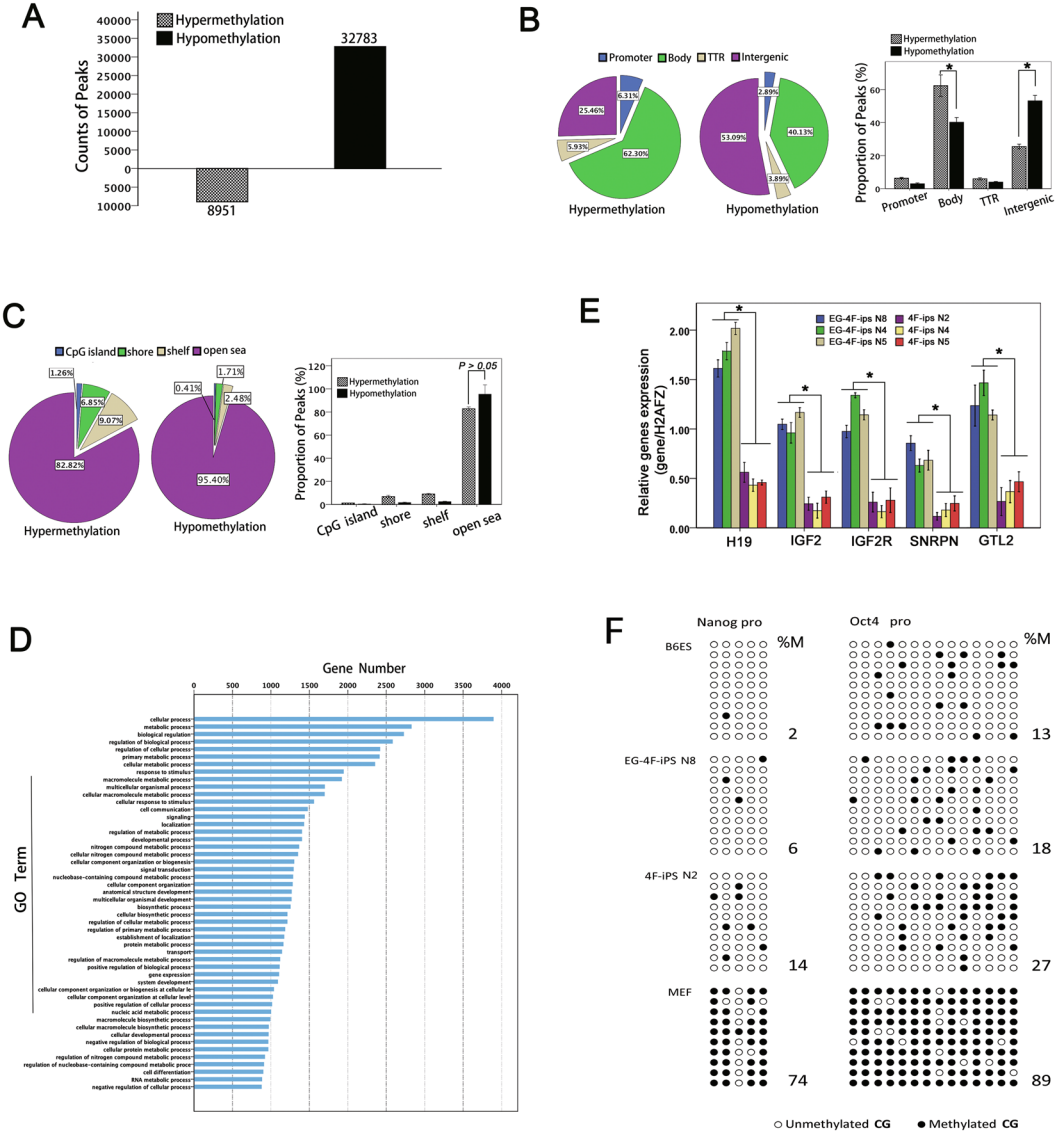
Genomic methylation patterns. (**A**) Hypo- and hypermethylation peak counts obtained from three cell lines in each group. EG-4F-iPSC versus 4F-iPSC peaks were considered as hypermethylation peaks; 4F-iPSC versus EG-4F-iPSC peaks were considered as hypomethylation peaks. (**B**) Average proportions of peaks within each region as defined by genomic structure. *P < 0.05 (**C**) Average proportions of peaks within each region as defined by distance from the CpG island. (**D**) Enrichment analysis of hypomethylated genes covered with peaks. (**E**) Comparison of imprinted gene expression in EG-4F-iPSCs and 4F-iPSCs as quantified by mRNA expression. Error bars indicate standard error of the mean (n = 3). Results were normalized to human histone H2A.Z (H2AFZ) expression. (**F**) Bisulfite sequencing of Nanog and OCT4 promoter region. Black circles represent methylated sites, white circles represent unmethylated sites. Global methylated cytosines are shown as %M.

We found changes in both hypo- and hypermethylation patterns took place throughout the genome between the two groups (Fig. 3A). The number of hypomethylated peaks was 32,783 in EG-4F-iPSCs, which was much higher than observed in 4F-iPSCs (8,951). This shows that EGC extracts could dramatically reduce methylation levels.

We then scrutinized DNA hypo- and hypermethylation distribution in the context of gene structure. Four relevant regions were defined: promoter, gene body, transcriptional termination region (TTR) and the intergenic region. DNA hypo- and hypermethylation were distributed differently in the four regions. As shown in Fig. 3B, hypermethylation was concentrated mainly within gene body areas and could also be detected in the intergenic region, suggesting EGC extracts could selectively induce different methylation modifications. Next, we examined methylation changes in four regions defined by their distance from CpG islands: the CpG island itself, Shore, Shelf and Open Sea regions; the latter three regions were 2 kb, 2–4 kb, and more than 4 kb from the CpG island, respectively. Most hypo- and hypermethylation patterns were detected in the Open Sea region. No differences in the distribution of hypo- and hypermethylation status were found in any of these four regions (Fig. 3C).

In addition, we considered hypomethylated genes with peak enrichment analysis (Fig. 3D). Differentially hypomethylated genes were primarily involved in cellular processes, metabolism, and biological processes.

Furthermore, demethylation of OCT4 and Nanog promotors was detected by bisulfite sequencing. As shown in Fig. 3F, the Nanog promoter region was highly methylated in MEFs (74%) and almost completely unmethylated in B6ES (2%). A similar methylation pattern was observed in OCT4 examined region, where 89% of the CpG sites were methylated in MEFs but only 13% were methylated in B6ES. EGCs extraction treatment (EG-4F-iPS N8) was shown to decrease the methylation levels of both regions (6% and 18%, respectively) than that of OSKM induction without EGCs treatment (14% and 27% respectively in 4F-iPS N2).

### Expression of imprinted genes increased after EGC extract treatment

Previous studies have demonstrated that methylation of imprinted genes could be erased during hybridization by fusion of thymic lymphocytes to EGCs^33^. To determine whether EGC extracts were also capable of eliciting epigenetic modifications on imprinted genes, we examined imprinted gene expression by real-time PCR. Figure 3E shows upregulation of both maternally expressed (Igf2r, Gtl2 and H19) and paternally expressed (Igf2, Snrpn) imprinted genes in EG-4F-iPSCs compared with 4F-iPSCs. Increasing expression of imprinted genes suggested exposure of MEF nuclei to EGC extracts could induced a more reprogrammable state.

### EGC extracts can partially reprogram MEFs

**Figure 4:**
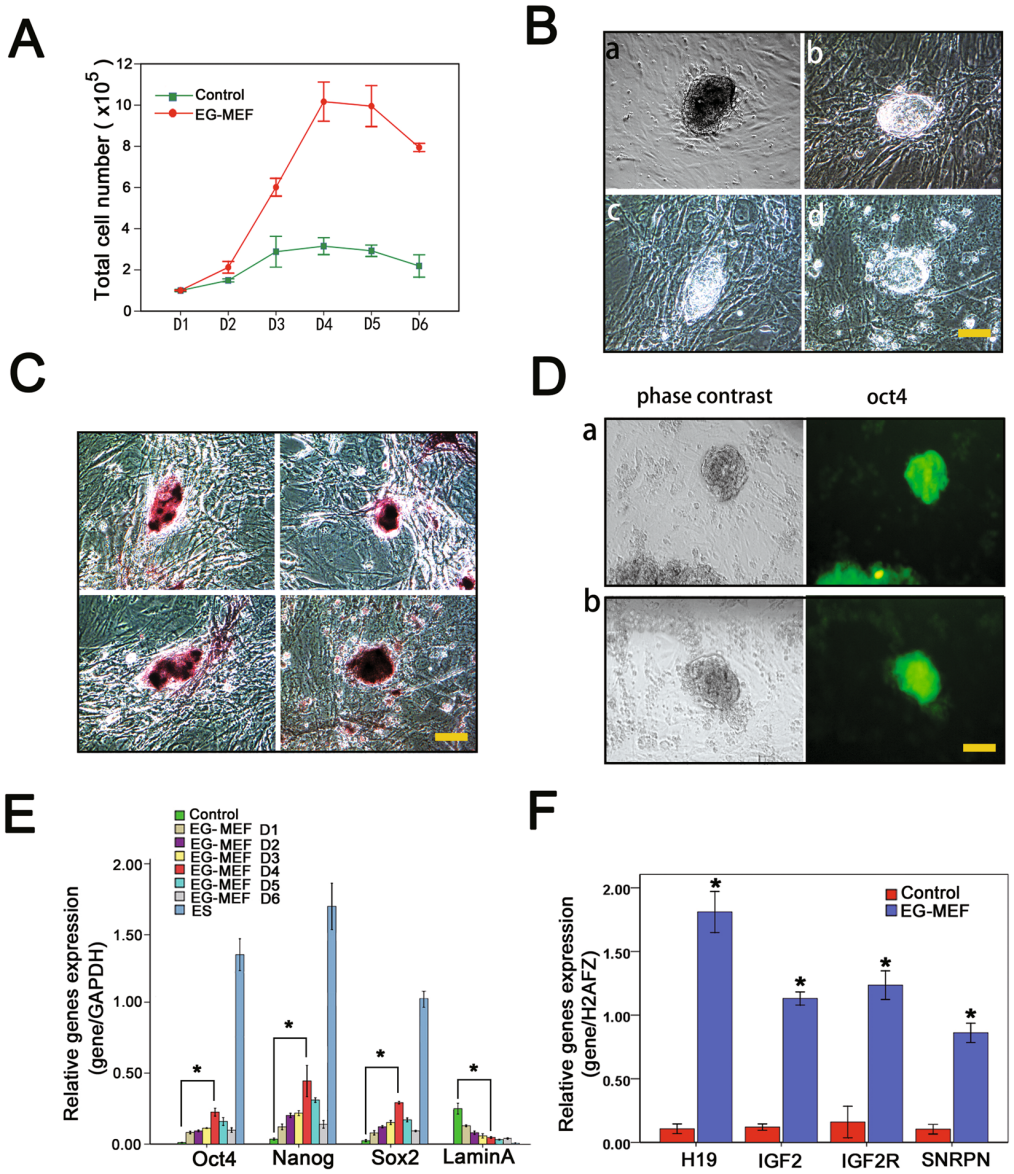
Reprogramming of permeabilized MEFs induced by EGC extracts. (**A**) Proliferation rate of EGC extract-treated MEFs compared with MEF extract-treated MEFs at different days. Error bars represent standard error of the mean (SEM; n = 3). (**B** a–d) Morphology of colonies formed following treatment of MEFs with EGC extracts. (**C**) Induction of alkaline phosphatase activity in MEFs after exposure to EGC extracts. (**D** a-b) Immunofluorescence staining of Oct4 positive colonies following exposure of MEFs to EGC extracts. Scale bars = 25 µm. (**E**) Quantitative PCR analysis of pluripotency marker and laminA expression in MEFs after incubation with EGC extracts. Error bars indicate SEM (n = 3). Results were normalized to GAPDH expression. *P < 0.05, versus control. (**F**) Q-PCR analysis of imprinted gene expression between EGC extract-treated MEFs and MEF extract-treated MEFs at 4 days after treatment. Results were normalized to human histone H2A.Z (H2AFZ) expression. *P < 0.05, **P < 0.01, versus control.

Because EGC extracts could enhance reprogramming of iPSCs with epigenetic modifications, we investigated the direct reprogramming roles of extracts on MEFs. We exposed reversibly permeabilized MEFs to EGC or MEF extracts (control). Cells were plated and the number of cells was determined from day 1 to day 6 after treatment, as shown in Fig. 4A–E. Our results indicate EGC extracts increase the growth rate of MEFs relative to the control, with a peak at day4 (Fig. 4A). Q-PCR analysis suggested that expression levels of Oct4, Nanog and Sox2 with extract treatment increased at first and then decreased, reaching a peak at day 4 consistent with observed changes in growth rate (Fig. 4E). Moreover, EGC extracts can induce the formation of a few colonies resembling ESC morphology as early as day 5 post-treatment (Fig. 4B), whereas, MEFs treated with MEF extracts did not show any morphological changes (data not shown). These colonies displayed positive AKP (Fig. 4C) and endogenous expression of Oct4 (Fig. 4D), but the colonies could not be passaged.

We next examined the expression of imprinted genes between MEF and EGC extract-treated cells by qPCR analysis. As shown in Fig. 4F, maternally expressed imprinted genes Igf2r and H19 were increased in EGC extract-treated cells, most especially H19, which exhibited remarkably higher expression. Moreover, paternally expressed imprinted genes Igf2 and Snrpn were also upregulated. These results demonstrate MEFs can be programmed by EGC extracts. Unfortunately, it is difficult to realize full reprogramming with extracts alone.

## Discussion

Our data indicate that EGC extracts improved reprogramming and facilitated the establishment of a pluripotent state in somatic cells. Experiments using somatic cell nuclear transfer^46^, cell fusion^36–38^ and extract-mediated^22,23,26^ induction confirmed that pluripotent cells, similar to oocytes, contain a lot of unknown factors capable of affecting somatic cell reprogramming. Previous reports have shown the roles of ESC, embryonal carcinoma and EGC extracts on reprogramming by hybrids with somatic cells; notably, the roles of EGCs in epigenetic reprogramming trumped the others^36,47,48^. Derived from PGCs, EGCs harbor an epigenome similar to that of PGCs, which show a genome-wide reprogramming of DNA methylation^34,35^; thus, EGCs might still contain demethylation factors and be better for reprogramming than ESCs. As such, we performed reprogramming using EGC extracts in the present study instead of ESC extracts.

As we expected, when OSKM induced MEFs were exposed to EGC extracts, we observed increased expression of pluripotent genes, accelerated appearance and number of ESC-like colonies, and significantly improved reprogramming efficiency. Previous achievement of reprogramming was restricted only to fusion of somatic cells and EGCs^22^. Here, we provide a new platform to improve reprogramming.

In addition, we found imprinted gene expression was also increased in EGC extract-treated cells. Genomic imprinting is essential for normal mammalian development and shows a substantial degree of stability in ESCs^49^. Further, abnormal allelic expression of imprinted genes in iPSCs has previously been shown to correlate with extensive methylation^43,44^, which could cause aberrant cell differentiation and obstruct transformation of iPSC lines to be used for regenerative medicine.

With further analysis of whole-genome methylation, we found that global demethylation was lower in EG-4F-iPSCs than in 4F-iPSCs. And EGC extraction treatment was shown to increase the methylation levels of both Nanog and OCT4 promoter regions. These were consistent with previous reports which showed that methylation of imprinted or non-imprinted genes was erased in EGC-fused cells^23^. Reversion of nuclei also occurred after fusion with ESCs; however, methylation patterns of imprinted genes were maintained^23,47^. This suggested EG-4F-iPSCs acquired improved pluripotency with less epigenetic memory.

Unfortunately, compared with EGC extract and OSKM combination, which efficiently induced fully competent iPSCs, application of EGC alone was ineffective at reprogramming of MEFs. Such a disparity may reflect the importance of varying reprogramming kinetics among different proteins. Apparently, amounts and effective time of proteins in the EGC extract may be inadequate and could not reach the reprogramming threshold.

The present study explores EGC extract as a unique reprogramming catalyst. Combining EGC extracts with OSKM resulted in improved efficiency and quality of generated iPSCs with less epigenetic memory as indicated by actively drive the DNA demethylation process. The reprogrammed cells tended to have more global hypomethylation. Use of EGC extracts in reprogramming may provides new approach and research strategies to yield more efficient and safer iPSCs. Further identification of relevant demethylation factors and reprogramming factors in the EGC extracts may provide us better understanding of the PGC demethylation mechanism and a significant advance in generating iPSCs in a safer manner.

## Materials and Methods

### EGC line

Animal handling followed the National Institutes of Health guidelines, and experimental procedures were approved by the Harbin Medical University Ethical Review Committee. Mouse fetuses from C57BL/6 J females mated with DBA/2 J males were collected at 12.5 days of pregnancy (E12.5). Female mice were sacrificed and fetuses were dissected away from their extraembryonic membranes. Genital ridges were dissociated from the mesonephros and dorsal walls of embryos. Genital ridge tissue was washed once with calcium-free Dulbecco’s phosphate-buffered saline (DPBS) and incubated in trypsin/EDTA solution for 5–10 min at 37 °C. Next, PGCs were dissociated by gently disrupting the genital ridge and the resulting cell suspension was centrifuged at 250 × g for 5 min. The cell pellet was resuspended in KnockOut™ Dulbecco’s Modified Eagle’s Media (DMEM; Gibco, USA) containing 20% KnockOut Serum Replacement (Gibco), 2 mM L-glutamine, 1% nonessential amino acids (NEAA), 0.1 mM 2-mercaptoethanol (Gibco), 20 ng/ml recombinant human basic fibroblast growth factor (Invitrogen, USA), 1000 U/ml recombinant leukemia inhibitory factor (LIF; Invitrogen), 40 ng/ml stem cell growth factor (Peprotech, USA), 1% penicillin and streptomycin (P/S; Gibco). After culture for 7−10 days, colonies were picked and seeded into new wells containing feeder cells for subculture. After 2–3 days, cells were passaged and cultured in LIF-containing medium.

### Cell culture

MEFs were isolated from E13.5 B6D2F1 mouse embryos and cultured in DMEM with 10% fetal bovine serum (FBS). R1 ESCs were cultured in DMEM with 15% ESC-qualified FBS (Invitrogen), 0.1 mM NEAA, 0.1 mM 2-mercaptoethanol and 1000 U/ml LIF. Plat-E cells (Cyagen, USA) were maintained in DMEM containing 10% FBS, 1% P/S, 1 mg/ml Puromycin (Sigma, USA) and 100 mg/ml blasticidin S (Merck, Germany).

### Cell extract preparation

EGCs were washed in DPBS and centrifuged at 1000 rpm for 5 min at 4 °C. Next, cells were suspended in cold DPBS containing 1 mM dithiothreitol, 0.1 mM phenylmethylsulfonyl fluoride and 0.1 mM protease inhibitor cocktail, and then incubated for 3 h on ice. After incubation, cells were repeatedly frozen and thawed in liquid nitrogen. Cell lysates were then centrifuged at 25,000 rpm for 15 min at 4 °C. The pH and osmotic pressure of the supernatant were then adjusted to physiological levels, before the protein concentration of each batch of supernatant was measured using an ultraviolet spectrophotometer (Eppendorf AG 22331, Hamburg, Germany). Finally, extracts were frozen in liquid nitrogen for future research.

### Permeabilization

MEFs were washed in Ca^2+^- and Mg^2+^- free DPBS, permeabilization cold transport buffer (110 mM potassium acetate, 5 mM sodium acetate, 2 mM magnesium acetate, 1 mM EGTA, 2 mM dithiothreitol, protease-inhibitor cocktail and 20 mM HEPES) containing 1–15 µg/ml digitonin and then placed on ice for 2 min. The permeabilization reaction was halted by adding 900 µl of transport buffer and cells were collected by centrifugation at 700 × g for 10 min at 4 °C. To estimate permeabilization efficiency (Supporting Information Fig. S2), treated cells were incubated with 10 µg/ml propidiumiodide (PI, Sigma) for 10 min. Permeabilization was assessed by monitoring uptake of a 10,000-molecular weight dextran Oregon green 488 (50 µg/mL; Invitrogen) and PI. To reseal plasma membranes, the cell suspension was diluted with DMEM containing 2 mM CaCl_2_.

### Induction of pluripotent stem cells by EGC extracts and OSKM

The retroviral pMX vector with OSKM cDNAs was transfected into Plat-E cells using Lipofectamine® LTX (Invitrogen). Supernatants were collected at 48 h and used to infect MEFs. The second round infection occurred at 72 h. The following day (day 0), MEFs were permeabilized and suspended in MEF or EGC extracts containing an ATP-regenerating system. After resealing plasma membranes, treated MEFs were reseeded on a 24-well plate and cultured in ESC medium (DMEM medium supplemented with15% ESC-qualified FBS, NEAA, P/S). On day 3, transfected MEFs were seeded on a feeder layer. On day 4, medium was replaced with KnockOut DMEM medium containing 15% KnockOut Serum Replacement, 0.1 mM NEAA, 0.1 mM β-mercaptoethanol and 1000U/ml LIF. iPSC colonies were picked, expanded and passaged using 0.25% trypsin/ethylenediaminetetraacetic acid (EDTA) every 2–3 days on feeder layers in ESC medium.

### *In vitro* and *in vivo* differentiation

The *in vitro* differentiation potential of iPSCs was evaluated by embryoid body (EB) formation. Cells were dissociated with trypsin and then cultured in low-attachment plates with ESC medium without LIF for 6 days. The differentiation capacity was examined by RT-PCR check of germ layer markers (exoderm: *pax6*, mesoderm: *eomes*, endoderm: *ttr*). For *in vivo* differentiation, cells were injected subcutaneously into nude mice (Vital River Laboratory Animal Technology Co. Ltd, China). Tumors were collected after 2 months and processed for paraffin sectioning, followed by hematoxylin and eosin staining.

### Alkaline phosphatase, immunostaining and karyotype analyses

Alkaline phosphatase (AKP) activity was detected with an AKP stain kit (Beyotime, China). For immunofluorescence analysis, cells were fixed in 4% paraformaldehyde 15 min and rinsed with 0.25% Triton X-100 in DPBS. After blocking, cells were incubated with Oct4 and SSEA1 antibodies (Santa Cruz Biotechnology, USA). Secondary antibodies were conjugated to fluorescein isothiocyanate (FITC; Santa Cruz Biotechnology) and nuclei were counter stained with Hoechst 33342 (Sigma).

For karyotype analysis, cells were incubated with colchicine for 1.5 h and then exposed to a hypotonic treatment with 0.56% KCl solution for 15 min. Fixation was performed with methanol and glacial acetic acid mixture (3:1). Finally, cell spreads were stained with Giemsa (Sigma) and separate metaphase karyotypes were counted.

### Real-time PCR and RT-PCR

Total RNA was extracted from cells with TRIzol® reagent (Invitrogen) according to the manufacturer’s protocol. cDNA synthesis was achieved using oligo (dT) and SuperScript® reverse transcriptase (Takara, Dalian, China). Real-time PCR was performed using TransStart™ Top Green qPCR Super Mix Kit (TransGen, China). Relative amount of gene expression was analyzed by the 2-dd Ct method^50^. RT-PCR was performed using High Capacity cDNA Reverse Transcription Kit (ABI, American). Primer sequences are listed in Supplemental Method Table 1.

### Whole-genome methylation analysis

DNA (3 μg) was sonicated at intensity 4 for 200 cycles per burst for 55 s in a Covaris S2. DNA fragments were end-repaired, ATP-tailed and adapter ligated with a Sample Preparation Kit (Illumina, USA). Next, DNA was recovered by AMPure® XP Beads and prepared for methylated DNA immunoprecipitation (MeDIP) using a Magnetic Methylated DNA Immunoprecipitation Kit (Diagenode, Belgium) according to the manufacturer’s protocol. After MeDIP, remaining DNA was PCR-amplified with sequencing primers and used for sequencing (Illumina HiSeq. 2500, USA). Raw reads were preprocessed using the FASTX-Toolkit. MeDIP-seq peaks were counted using MACS. EG-4F-iPSC versus 4F-iPSC (control) peaks were considered as hypermethylation peaks; whereas, EG-4F-iPSC versus 4F-iPSC (control) negative peaks were considered as hypomethylation peaks. Genes covered by peak-enrichment analysis were assessed using GO stats software (Bioconductor).

### Bisulfite treatment and bisulfite sequencing

Demethylation of OCT4 and Nanog promotors was detected by bisulfite sequencing, in accordance with the method of Christel T. *et al*.^26^ Converted DNA were amplified by PCR using the primers listed below. The annealing temperatures were 60 °C. The PCR products were the cloned into the pGEM-T Easy Vector (Prpmega) system for sequencing. Primer pairs used were: OCT4 forward: 5′-GAGGTGCAATGGCTGTCTTGT-3′, reverse: 5′-ACCAACCAGTTGCTCGGATGC-3′; Nanog forward: 5′-GGTAGGGTAGGAGGTTTGAGG-3′, reverse: 5′-AAGGTTTTAGGCAACAATTAA-3′.

### Statistical analysis

All data were obtained from at least three independent experiments. Statistical analysis of the data was performed with a Student’s t-test. P < 0.05 was considered statistically significant. All data are shown as mean ± standard deviation.

## Electronic supplementary material

Supplementary Information
