# Supplementary material for: Embryonic germ cell extracts erase imprinted genes and improve the efficiency of induced pluripotent stem cells
Source: Sci Rep. 2018 Jul 19;8:10955. doi: 10.1038/s41598-018-29339-0 (PMC6053380; doi:10.1038/s41598-018-29339-0)
Supplement: Supplementary file 1 — Supplementary Information [file 41598_2018_29339_MOESM1_ESM.docx]

**Embryonic germ cell extracts erase imprinted genes and improve the efficiency of induced pluripotent stem cells**

Jing Hu^1,2^, Qiaoshi Zhao^1^, Yukuan Feng^3^, Na Li^1^, Yanli Gu^1^, Ruizhen Sun^1^, Lian Duan^1^, Yanshuang Wu^1^, Zhiyan Shan^1^ & Lei Lei^1^

^1^Department of Histology and Embryology, Harbin Medical University, Harbin, 150086, P.R.China. ^2^Department of Histology and Embryology, Mudanjiang Medical University, Mudanjiang, 157011, P.R.China. ^3^Key Laboratory of Tumor Prevention and Treatment of Heilongjiang Province, Mudanjiang Medical University, Mudanjiang, 157011,

Correspondence and requests for materials should be addressed to L. L. (email: [lei086@ems.hrbmu.edu.cn](mailto:lei086@ems.hrbmu.edu.cn)) or Z.Y. S. (email: [shanzhiyan1979@126.com](mailto:shanzhiyan1979@126.com))

**Running Title:** EGC extracts improve the efficiency of iPS cells

**Supporting Information**

**Table 1. Primer sequences for real-time PCR and RT-PCR**

| Gene | Forward primer (5′ to 3′) | Reverse primer (5′ to 3′) |
| --- | --- | --- |
| H19 (real-time PCR) | ctcctccccctaccttgaac | ccttggagcagattcctgag |
| Gtl2 (real-time PCR) | ttgaagcttggaaagccagt | cagcccatggtatcacacag |
| Igf2r (real-time PCR) | gcaccaagatgaagcagtca | acatccggtagctgttggtc |
| Ascl2 (real-time PCR) | ctcgtcggaggaaagca | caggagtcacccagggat |
| Tss3 (real-time PCR) | agacctccgacgagatcctt | ccttgaggatggagtggaaa |
| Dppa5 (real-time PCR) | ccgaagacctgaaagatcca | gactggagcatccacttggt |
| Igf2 (real-time PCR) | gtcgatgttggtgcttctca | aagcagcactcttccacgat |
| Snrpn (real-time PCR) | gtggggagaacttggtttca | aggagcctggggaataggta |
| Nnat (real-time PCR) | ccggcagaactgctcatcat | cagcttctgcagggagtacc |
| Ins2 (real-time PCR) | tgtcccgccgtgaagt | gtgccaaggtctgaaggt |
| Mest (real-time PCR) | cagaaccgcagaatcaacct | cgtctttgaggagcttttgg |
| Oct4 (real-time PCR) | agttggcgtggagactttgc | cagggctttcatgtcctgg |
| Nanog (real-time PCR) | tcttcctggtccccacagttt | gcaagaatagttctcgggatgaa |
| Sox2 (real-time PCR) | cacaactcggagatcagcaa | ctccgggaagcgtgtactta |
| Lamin A (real-time PCR) | ggatgctgagaacaggctaca | ctctcgctgcttcccgttatc |
| GAPDH (real-time PCR) | aggtcggtgtgaacggatttg | tgtagaccatgtagttgaggtca |
| H2afz (real-time PCR) | ctgaagtagtgggttttgattg | gggatatgacctttattgagct |
| Pax6 (RT-PCR) | agacttaaccaagggcggt | tagccaggttgcgaagaact |
| TTR (RT-PCR) | agtcctggatgctgtccgag | tcctgagctgctaacacgg |
| Eomes (RT-PCR) | caacataaacggactcaacc | cgacctccagggacaatct |
| Gapdh (RT-PCR) | tgaaggtcggtgtgaacggat | cagggggctaagcagttggt |


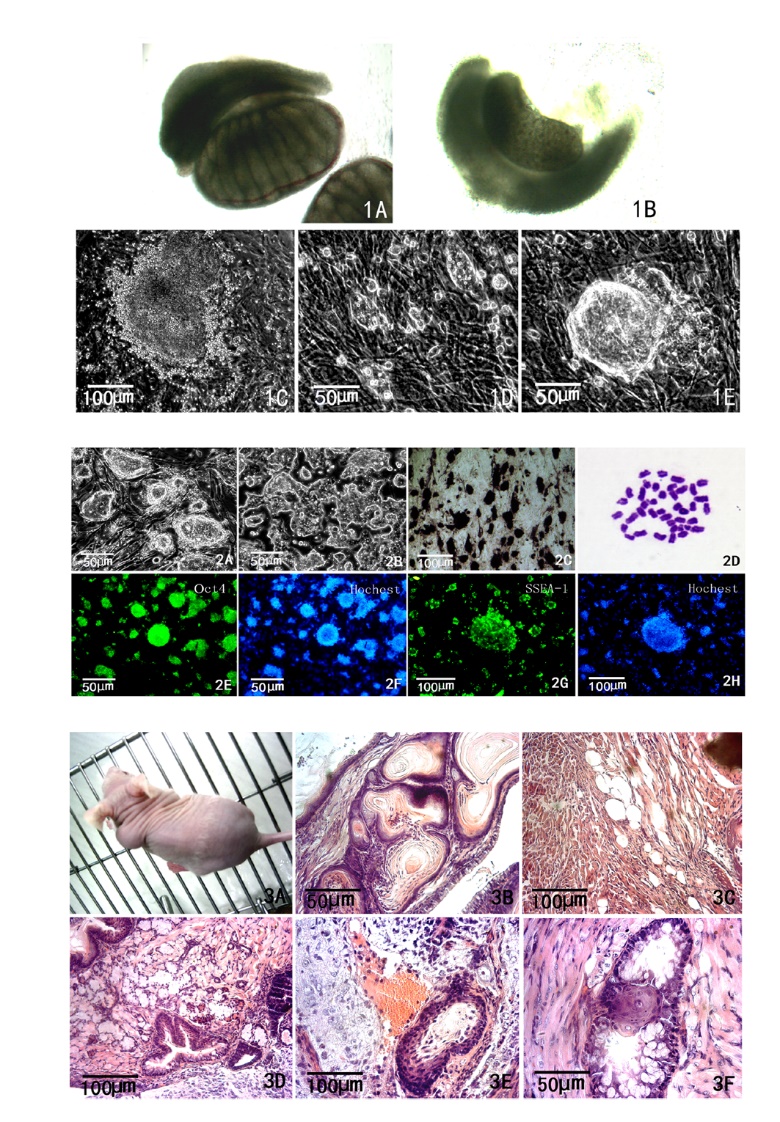


**S1 Fig.** Establishment of embryonic germ (EGCs). (1A) Male genital ridge. (1B) Female genital ridge. (1C) Primordial germ cells after 1 day of primary culture. (1D–E) Morphology of colonies after 3–4 (1D) and 7–10 (1E) days of primary culture. Identification of EGCs. (2A-B) Morphology of EGCs cultured on a feeder layer (2A) or gelatin (2B). (2C) Alkaline phosphatase stain, presenting as typical dark brown. (2D) Karyotype analysis (20 pairs). (2E-H) Immunocytochemistry for OCT4 (2E), SSEA-1(2G), and Hoechst 33342 cell nucleus stain (2F, 2H). Differentiation of EGCs *in vivo*. (3A) Nude mouse after 6 weeks of EGC injections (black arrow indicates resulting teratoma). (3B) Epidermis (ectodermal structure). (3C) Skeletal muscle and fat (mesodermal structures). (3D) Digestive gland endothelium (endodermal structure). (3E) Blood (mesodermal structure) and nerve epithelium (ectodermal structure). (3F) Respiratory epithelium (endodermal structure).


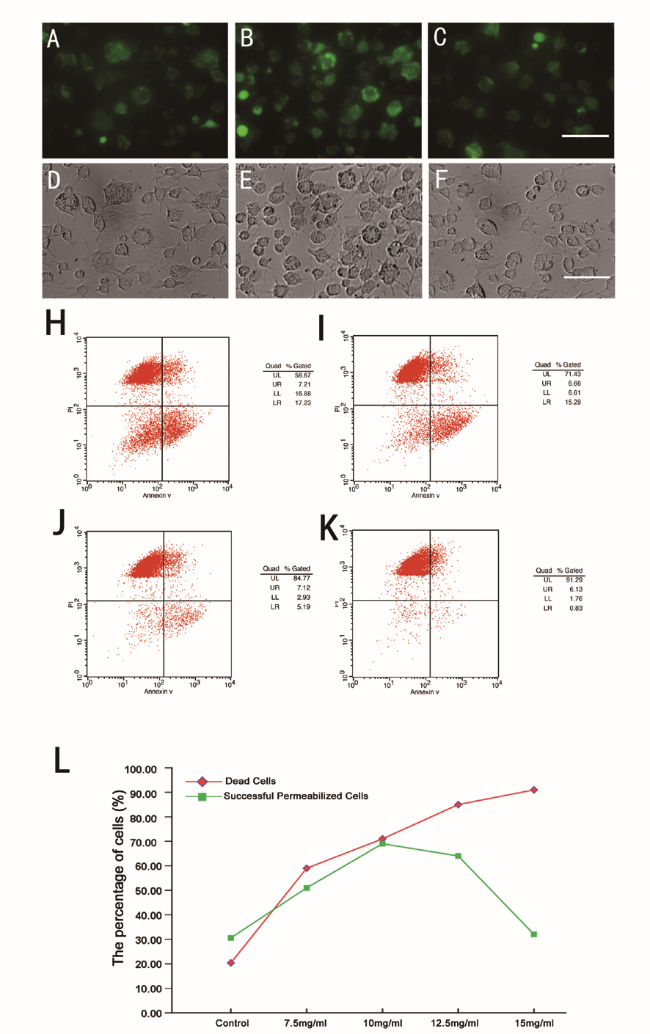


**S2 Fig.** Optimal permeabilization concentration of digitonin. (A–C) Fluorescence staining of mouse embryonic fibroblasts (MEFs) permeabilized with digitonin at a concentration of 7.5 (A), 10 (B), or 12.5 µg/mL (C), as assessed by uptake of the dextran Oregon green 488, 10,000 MW. Scale bars = 25 µm. (D–F) Light microscopy photographs of A–C, respectively. Scale bars = 25 µm. (G–J) Results detected by FACS: UL, cells died; LL+LR, cells were successfully permeabilized by digitonin. MEFs permeabilized with digitonin at a concentration of 7.5 µg/mL (G), 10 µg/mL (H), 12.5 µg/mL (I), or 15 µg/mL (J). (L) Percentage of permeabilized cells treated with different doses of digitonin. Most cells (69%) were permeabilized at 10 µg/mL and less dead cells were observed.

**

**

**S3 Fig.** The full-length gels of RT-PCR.
